# Lessons Unlearned: The Barriers to Lyme Borreliosis Vaccination and the Risk of Repeating History

**DOI:** 10.3390/vaccines14080698

**Published:** 2026-08-12

**Authors:** Deepak Nagra, Kathryn Biddle, Katie Bechman, Victoria Allen, James Galloway

**Affiliations:** Centre For Rheumatic Disease, King’s College London, London SE5 9RS, UK

**Keywords:** lyme disease, borrelia burgdorferi, LYMErix, VLA15, PF-07307405, OspA, vaccine safety, VAERS, pharmacovigilance, vaccine hesitancy, post-infectious lyme arthritis, risk perception

## Abstract

Lyme borreliosis is the most common vector-borne disease in the temperate Northern Hemisphere, with approximately 476,000 diagnoses annually in the United States and over 130,000 reported cases across Europe each year, figures that substantially underestimate true incidence. In 1998, LYMErix became the first licensed vaccine against Lyme borreliosis. Its withdrawal four years later was not driven by evidence of vaccine failure but by the collapse of public confidence, and regulatory endorsement that any successful vaccine programme requires. The field was without a licensed product for twenty-four years. VLA15 (now designated PF-07307405), a second-generation recombinant outer surface protein A vaccine, is now at regulatory submission following phase 3 results reported in March 2026. We examine the barriers that led to LYMErix’s withdrawal, revisit the evidence that generated and sustained the safety controversy and consider whether the conditions now exist for a different outcome. The lessons for a robust post-marketing surveillance programme extend beyond Lyme borreliosis to broader questions about how modern medicine evaluates vaccine risk, interprets safety signals, and communicates under uncertainty.

## 1. Introduction

Vaccination against Lyme borreliosis has re-emerged as a highly topical issue following completion of the phase 3 VALOR clinical trial program in March 2026 [1]. Through this article we revisit the history of the LYMErix vaccine and discuss the need for a vaccine against Lyme borreliosis with a spotlight on the results of the recently concluded phase 3 trial of the VLA15 Lyme vaccine.

Regulatory submissions to the US Food and Drug Administration and European regulators are anticipated. If licensed, VLA15, now designated PF-07307405, would become the first vaccine against Lyme borreliosis available in over two decades, following withdrawal of LYMErix from the market in 2002 [2].

The return of a vaccine against Lyme borreliosis is therefore not simply the arrival of a new vaccine candidate. It is a test of whether the medical and scientific community has learned from one of the most instructive and controversial episodes in modern vaccinology.

If history is to avoid repeating itself, the lessons from LYMErix must be understood not only within the context of Lyme borreliosis but across the wider vaccination field.

## 2. Why Lyme Borreliosis Matters

Lyme borreliosis is the most common vector-borne disease in the temperate Northern Hemisphere [3]. In the United States, recent estimates suggest approximately 476,000 diagnoses annually, a figure derived from insurance claims and physician survey data that substantially exceeds the 63,000 cases formally notified through passive surveillance [4]. European surveillance data report approximately 130,000 cases per year across 25 countries, though this is almost certainly a substantial underestimate given inconsistent reporting systems and variable case definitions; true incidence is estimated to be two to ten times higher in most countries [5].

Within Europe, the greatest burden is observed in the Baltic states, Slovenia, and parts of central Europe, though the epidemiology is changing [6]. There is increasing evidence that the geographic range of *Ixodes* tick vectors is expanding northwards, driven in part by changing climatic conditions, with measurable altitudinal and latitudinal shifts documented in Scandinavia and central Europe [7,8]. The implication is that populations currently at modest risk will face higher exposure over coming decades.

The causative bacterium, *Borrelia burgdorferi* sensu lato is a tick-borne spirochete and encompasses more than 18 genospecies, of which three commonly infect humans. *B. burgdorferi* sensu stricto predominates in North America; *B. afzelii* and *B. garinii* account for most European disease, with differing clinical tropisms that have direct implications for vaccine design [9].

Lyme borreliosis is traditionally described as progressing through three clinical stages, early localized, early disseminated, and late disease, although these stages often overlap, and only a minority of untreated patients progress to late manifestations. Approximately 90% of patients present with early localized disease, most commonly as erythema migrans, a gradually expanding erythematous rash at the site of the tick bite, often with an annular or “bull’s-eye” appearance, and this may be accompanied by flu-like symptoms such as myalgia and arthralgia [10]. Notably the bull’s-eye appearance is not the only clinical appearance [11]. Up to 20% present with early disseminated disease, which occurs when *B. burgdorferi* spreads via the bloodstream from the initial site of infection to other tissues and organ systems, causing manifestations including neuroborreliosis, carditis, arthritis, or multiple erythema migrans lesions [12]. Late disease develops in only a minority of untreated or inadequately treated patients and is characterized primarily by recurrent or persistent arthritis, most commonly affecting large joints, with less frequent chronic neurological complications [13].

The importance of Lyme borreliosis extends beyond the acute infectious episode which can present with arthralgias, myalgias and flu-like symptoms [14]. Although most cases respond to antibiotic therapy, a subset of patients develop post-infectious Lyme arthritis, a persistent synovitis that continues despite microbiologically adequate treatment [15,16]. The pathogenesis is a dysregulated, self-sustaining inflammatory response that persists after spirochetal killing. Affected patients require disease-modifying therapy, including methotrexate and tumor necrosis factor inhibitors, and the clinical picture is indistinguishable from seronegative inflammatory arthritis [17,18,19]. Erosive joint damage occurs in a minority [20]. Early antibiotic treatment is effective but depends on timely diagnosis in the early stages of disease, which in the absence of a reliable point-of-care test remains imperfect [21].

Lyme-associated inflammatory arthritis can be considered, at least in principle, a preventable form of chronic inflammatory disease.

## 3. The Asymmetry of Vaccine Perception

Any discussion of Lyme vaccination must recognize a fundamental asymmetry in how vaccines are perceived by clinicians and recipients. Understanding this asymmetry is central to understanding why LYMErix failed and why VLA15 faces comparable risks.

For clinicians, risk perception is shaped by direct exposure to disease. Physicians encounter facial nerve palsies, meningoradiculitis, carditis, and inflammatory arthritis requiring immunosuppressive therapy. Vaccines prevent morbidity and mortality from various infections. By contrast, serious vaccine-related adverse events are rare and are unlikely to be encountered directly during routine clinical practice [22,23].

The vaccine recipient experiences the calculation very differently. Most individuals considering vaccination against Lyme borreliosis will never have seen a case of the disease. Most will never develop it even without vaccination, with the likelihood of Lyme borreliosis after a tick bite being under 2% [24]. The benefit is therefore probabilistic, deferred, and invisible: the disease that does not occur leaves no trace. In contrast the risks of a vaccine are immediate and embodied: the injection, the local reaction, and the systemic symptoms that may follow. Concepts such as herd immunity or population-level protection remain statistical abstractions compared with the immediacy of a potential adverse event [25]. Lyme borreliosis adds a further complication: because it is not transmitted person-to-person, there is no herd immunity benefit, and the case for vaccination rests entirely on individual protection.

These perceptual differences are amplified by well-characterized cognitive mechanisms [26,27]. Omission bias, the tendency to judge harm caused by action as morally worse than equivalent harm caused by inaction, has been specifically documented in vaccination decisions: individuals show reluctance to vaccinate when the vaccine itself carries a risk, even when that risk is substantially lower than the risk of the disease prevented [28]. The availability heuristic compounds this: people estimate the probability of an event by the ease with which examples can be recalled, so that widely publicized reports of adverse events following vaccination can lead individuals to overestimate the true frequency of these events relative to their actual incidence [29]. A notable example from recent times includes thromboembolic events and transverse myelitis from the COVID-19 vaccination campaign which despite reports, remains an infrequent event [30,31]. Vaccines are uniquely vulnerable to the paradox of preventive medicine: when they succeed, the diseases they prevent become less visible, while potential harms associated with vaccination become more psychologically salient [32]. The near disappearance of paralytic polio in many high-income countries, for example, has left few living memories of the disease’s devastating consequences, while anxieties surrounding vaccine safety remain highly visible and emotionally resonant. Experimental evidence confirms that brief exposure to online content highlighting vaccine harm negatively impacts vaccination intentions and that vaccine-critical narratives propagate more effectively through social networks than vaccine-supporting evidence [33].

This divergence in perspective became particularly visible during the COVID-19 pandemic. Vaccine-induced immune thrombotic thrombocytopenia emerged as a genuine, mechanistically characterized adverse event associated with adenoviral vector vaccines, affecting approximately one to two individuals per 250,000 doses [34]. Critically, adverse events were concentrated in younger adults, the population least likely to derive major individual benefit from vaccination against severe COVID-19 outcomes. The benefit was real, with a reduction in rates of infection, hospitalization and mortality; however, it accrued predominantly to a different and older population. The recognition of vaccine induced immune thrombotic thrombocytopenia associated with adenoviral-vector vaccines, particularly among younger adults, may have contributed to hesitancy that subsequently extended to mRNA vaccines, for which the same safety signal had not been demonstrated. The resulting fall of public confidence ultimately had effects that extended far beyond the direct morbidity attributable to the adverse event itself [35].

A rhetorical analogy rather than evidence-based comparison that describes this situation is similar to a passenger boarding an airplane who experiences some version of that transient awareness of risk. Rationally, the probability of harm is extraordinarily small, yet the thought is psychologically vivid. Vaccination asks something structurally similar: accept a certain, immediate, visible intervention to prevent a probabilistic harm that will most likely never arrive [36]. The psychological challenge is real and should not be dismissed.

Crucially, the vaccine industry has not been blameless in contributing to the environment of skepticism that new vaccine programmes now face. RotaShield, a rotavirus vaccine, was withdrawn in 1999 following a genuine post-marketing signal of intussusception [37]. Dengvaxia increased the risk of severe dengue in seronegative recipients through antibody-dependent enhancement, a genuine subgroup harm that was identifiable in the existing trial data but not communicated to regulators or the public in a timely manner, with devastating consequences for broader vaccine confidence in the Philippines [38,39,40]. The medical community cannot dismiss safety concerns simply because later evidence disproves them, and it cannot assume that strong efficacy data alone will determine public acceptance.

## 4. The Rise and Fall of LYMErix

With this context established, the LYMErix story can be read not as an isolated episode of public irrationality but as a case study on what happens when the structural vulnerabilities described above converge with specific scientific and commercial circumstances.

LYMErix, developed by SmithKline Beecham, was evaluated in a phase 3 trial involving 10,936 participants across 31 US sites, demonstrating efficacy of 76% after three doses when compared to placebo [41]. Amongst the trial population there were 66 confirmed cases of Lyme borreliosis in the placebo group when compared to 16 cases in the vaccine administered group. Clinical efficacy was defined as having the presence of infection through polymerase chain reaction (PCR) testing for Lyme borreliosis at the time of suspected symptoms attributable to Lyme borreliosis, clinical symptoms and antibody titers up to 20 months after vaccine administration. All of the study sites and participants were within the United States of America. A contemporaneous trial of the ImuLyme, developed by Pasteur Merieux Connaught, enrolling 10,305 participants, reported efficacy approaching 92% in per-protocol analyses [42]. Both trials showed acceptable safety profiles, with no significant difference in serious adverse events between vaccine and placebo recipients. The FDA licensed LYMErix in December 1998.

The regulatory positioning proved important from the outset. The Advisory Committee on Immunization Practices (ACIP) issued a permissive recommendation in June 1999, advising that vaccination “should be considered” for individuals in endemic areas with significant exposure risk, rather than making a routine population-wide recommendation. This distinction had material consequences. In the absence of a routine ACIP recommendation, LYMErix was not incorporated into the US Vaccine Injury Compensation Program, the no-fault system that provides compensation mechanisms and partial liability protection for vaccine manufacturers. Consequently, alleged vaccine injuries would be pursued through conventional civil litigation, increasing the commercial and legal vulnerability of the programme from the outset ImuLyme, notably, was never submitted for regulatory approval. The decision was not driven by trial failure but by concerns generated by contemporaneous laboratory research and the rapidly deteriorating commercial and regulatory climate surrounding vaccination against Lyme borreliosis. The consequences for the field were profound, with the withdrawal of a vaccine which could reduce morbidity and associated socio-economic impact of Lyme Borreliosis, and, in many respects, as consequential as the subsequent withdrawal of LYMErix itself [43].

The safety controversy surrounding LYMErix originated in a three-page Report published in *Science* in July 1998, coinciding almost exactly with FDA approval. Gross and colleagues identified partial sequence homology between an immunodominant epitope of outer surface protein A (OspA) and a peptide of human leucocyte function-associated antigen 1 (hLFA-1) and demonstrated that synovial fluid T cells from patients with antibiotic-refractory Lyme arthritis produced interferon-gamma in response to both [44]. These findings raised the possibility that immune responses directed against OspA might cross-react with host tissue through molecular mimicry, potentially triggering autoimmune arthritis in genetically susceptible individuals, particularly carriers of HLA-DRB1*0401. Although mechanistic and preliminary, the hypothesis was biologically plausible, highly specific and published in one of the world’s most influential scientific journals at the precise moment the first vaccine against Lyme borreliosis entered public use.

The evidentiary basis of this hypothesis warrants close examination. The human data comprised eleven patients with antibiotic-refractory Lyme arthritis and nine controls, all of whom had established inflammatory arthritis; there was no healthy control group. The sequence homology consisted of six matching amino acids out of nine, a degree of identity not unusual across large protein databases. Most significantly, a methodological limitation acknowledged by the original investigators substantially weakens the study’s central finding. Owing to technical limitations in the purification of hLFA-1, insufficient quantities of the protein were available for testing, and hLFA-1 was therefore evaluated at a molar concentration approximately three orders of magnitude lower than OspA. This disparity meant that T-cell responses were initially compared under non-equivalent experimental conditions that favored detection of stronger responses to OspA. When the authors subsequently compared equimolar concentrations of the two antigens, the T-cell response to OspA decreased to levels comparable with those elicited by hLFA-1, whereas no meaningful difference between the antigens remained. Thus, the apparent disparity observed in the original experiments reflected differences in antigen concentration rather than intrinsic differences in T-cell reactivity. These findings substantially weaken the evidence for biologically meaningful molecular mimicry, as the proposed cross-reactivity was demonstrated under experimental conditions that systematically favored the observed effect [44].

The authorship and timing of this paper are relevant to its interpretation. Allen Steere, the principal investigator of the SmithKline Beecham phase 3 efficacy trial, was a co-author of the *Science* report proposing that the vaccine’s active ingredient might cause treatment-resistant arthritis; his LYMErix trial efficacy results appeared in the *New England Journal of Medicine* the same week [41,44]. Whether this represented principled disclosure of a potential safety concern now of regulatory attention, or whether a hypothesis resting on eleven patients warranted the authority conferred by a *Science* Report, remains a matter of interpretation. What is not in dispute is that the simultaneous publication of efficacy and safety concern by the same investigator, in journals of the highest possible visibility, was consequential.

Subsequent investigation did not support the hypothesis that OspA vaccination could induce autoimmune arthritis through molecular mimicry and cross-reactivity with hLFA-1 in genetically susceptible individuals. Trollmo and colleagues demonstrated that the hLFA-1 peptide functioned only as a weak partial agonist at the relevant T cell receptor, substantially undermining the mechanistic basis of the molecular mimicry model [45]. The FDA Vaccines and Related Biological Products Advisory Committee reviewed post-marketing safety data in January 2001 and found no causal relationship between LYMErix vaccination and arthritis [2]. Most definitively, a case–control study published by Ball and colleagues in 2009 enrolled individuals in whom arthritis had developed after LYMErix vaccination alongside three comparator groups, performed full HLA-DRB1 allele typing, and found no excess of treatment-resistant Lyme arthritis alleles in the vaccine-exposed group [46]. The authors concluded that LYMErix was not a major factor in the development of arthritis in these individuals. The vaccine that had been withdrawn seven years earlier was, by any evidential standard, exonerated.

The post-marketing surveillance data that sustained the safety narrative in public and legal discourse during this period illustrate a parallel challenge, namely the limitations of spontaneous pharmacovigilance. Between December 1998 and July 2000, 905 adverse event reports following LYMErix vaccination were submitted to the Vaccine Adverse Event Reporting System (VAERS) across approximately 1.4 million distributed doses, of which 59 were coded as arthritis-related events [47]. These reports were widely interpreted as evidence of vaccine-induced harm [48,49].

VAERS is a signal-generating system. Reports are submitted voluntarily following any event occurring temporally after vaccination, with no denominator, no comparator population, and no capacity to distinguish vaccine-attributable events from those that would have occurred regardless of vaccination. The Lathrop analysis, conducted by CDC and FDA scientists, calculated that background arthritis incidence in an unvaccinated population of this size would predict over 2000 arthritis events over the same period [47]. The 59 reports were far below expected background incidence, not above it. There was no temporal clustering of arthritis events following individual doses, as would be expected for an immune-mediated mechanism. The data, correctly interpreted, provided no support for the concern, with the understanding that spontaneous reporting is subject to underreporting.

A specific feature of the VAERS data warrants attention. A subgroup of ten individuals included their HLA-DR4 status within their adverse event submissions, of whom 50% had serious events. A “serious event” reported to VAERS is defined as one which resulted in life-threatening illness, hospitalization, prolongation of hospitalization, persistent or significant disability/incapacity, or death, or required an intervention to prevent any of these events [50]. Among these ten patients, the serious events were death in four patients due to hypertensive cardiovascular disease in two patients, myelofibrosis in one patient and suicide due to musculoskeletal discomfort in one patient. Other serious events were reported as arthritis, neurological insult such as cerebrovascular ischemia, demyelinating disease and aseptic meningitis.

The LYMErix controversy did not occur in isolation. In 1998, a paper in *The Lancet* proposed a link between MMR vaccination and autism, later retracted but not until 2010. In 1999, the RotaShield rotavirus vaccine was withdrawn for a genuine intussusception signal at approximately one to two cases per 10,000 doses [37]. Public concern about vaccines was already heightened, and the broader climate made LYMErix-related fears more credible than the underlying evidence warranted.

In December 1999, a class action lawsuit was filed on behalf of approximately 400 individuals attributing arthritis to LYMErix vaccination. Although the FDA concluded in January 2001 that evidence did not support a causal relationship, and despite the subsequent failure to reproduce the proposed immunological mechanism, commercial viability had collapsed. GlaxoSmithKline withdrew LYMErix in February 2002, citing poor consumer demand. The legal settlement that followed in 2003 covered legal fees only; no compensation was awarded for vaccine injury claims [2,51,52]. A potentially effective vaccine against Lyme borreliosis disappeared for over twenty years. ImuLyme, which had demonstrated comparable efficacy in phase 3 trials, was never submitted for licensure. Its withdrawal before approval illustrated how a climate of concern can suppress a second product that never faced a single regulatory reviewer [53].

## 5. The Arrival of VLA15

VLA15, now designated PF-07307405, represents a substantially different vaccine from LYMErix, and the scientific advances are specific rather than incremental.

Where LYMErix was a monovalent construct covering only *B. burgdorferi* sensu stricto OspA serotype 1, VLA15 is hexavalent, incorporating OspA serotypes 1 to 6 to provide broader geographic and genospecies coverage across both North American and European disease [54]. Critically, the construct excludes the N-terminal region of the serotype 1 antigen implicated in the molecular mimicry hypothesis, substituting it with the corresponding sequence from serotype 2 [54,55]. Engineered disulphide bonds further stabilize the conformational epitopes. Notably, the same laboratory group that proposed the hLFA-1 hypothesis in 1998 published the modified OspA antigen designed to eliminate this theoretical risk in 2004 [55]; this modified antigen became the structural basis of VLA15. More than two decades of post-withdrawal observation have produced no clinical evidence that recombinant OspA vaccination induces cross-reactive autoimmune arthritis in humans [56]. The underlying mechanism of protection is unchanged: anti-OspA IgG enters the tick during feeding and neutralizes spirochaetes in the midgut before transmission to the human host.

Phase 1 evaluation demonstrated acceptable safety and immunogenicity across dose levels in healthy adults [57]. Phase 2 trials across adults, adolescents, and children aged five and above showed robust anti-OspA IgG responses across all six serotypes with no vaccine-related serious adverse events [58]. The VALOR phase 3 trial enrolled 9437 participants aged five and above, randomized 1:1 to VLA15 or placebo across endemic sites in the United States, Canada, and Europe. Topline results reported in March 2026 demonstrated vaccine efficacy of 73.2% from 28 days after the fourth dose (95% CI 15.8 to 93.5) [59].

The inclusion criteria for the trial were limited to participants who reside in areas with endemic Lyme borreliosis and excluded those who were pregnant, had a history of Lyme carditis, neuroborreliosis, arthritis, disseminated disease or a tick bite within 4 weeks of trial participation. Outcomes were a combination of geometric mean ratio of OspA titers, adverse events and relative incidence rate reduction in confirmed Lyme borreliosis when compared to placebo for a period of 12 months.

The trial is not without important limitations, and they require honest acknowledgement. Fewer confirmed Lyme borreliosis cases accrued over the study period than projected, and the pre-determined statistical criterion for the primary analysis, requiring the 95% confidence interval lower bound to exceed 20%, was not met, a requirement for success of the primary analysis. The criterion was met in the second pre-specified analysis, and the point estimate of 73.2% is meaningful and consistent with the LYMErix phase 3 programme. The results are sponsor-reported, awaiting a peer-reviewed publication. Two further factors contributed to the lower case accrual: below-average tick activity during the study period and the early discontinuation of a substantial proportion of enrolled participants following protocol violations identified at US study sites in February 2023 [59]. No vaccine-related serious adverse events were identified. Regulatory submissions are anticipated in 2026.

The wide confidence interval will attract adversarial characterization in public and media discourse. A pre-emptive and technically accurate explanation of why the confidence interval is wide, grounded in the specific circumstances of case accrual and participant disenrollment, is an essential component of the communication strategy for this programme. The LYMErix experience demonstrated clearly that once a safety or efficacy narrative becomes established in the public domain, correction requires substantially more effort than prevention.

It is worth noting briefly that VLA15 is not the only approach in development. Moderna has mRNA-based OspA vaccine candidates in early clinical evaluation, and a single-dose long-acting anti-OspA monoclonal antibody, TNX-4800, that directly provides anti-*Borrelia* immunity has completed Phase 1 evaluation [60,61]. In this study, 44 patients were randomized to receive a subcutaneous injection of either 0.5, 1.5, 5 or 10 mg/kg of TNX-4800 or placebo (NCT0486328) with all study sites in Nebraska, USA. Early data have suggested a robust response up to 12 months after administration without the need for further booster dosing within this time frame. Passive immunization bypasses the requirement for active immunogenicity and may be particularly relevant for immunosuppressed patients, those receiving biologic therapies, JAK inhibitors, or B-cell depleting agents, in whom VLA15 antibody responses may be attenuated and who have not been specifically studied in published trial data. These approaches are complementary rather than competing.

## 6. Beyond Lyme Borreliosis

Licensure alone will not determine the success of vaccination against Lyme borreliosis. The critical question is whether clinicians, public health authorities, and patients interpret the evidence in the same way, and whether the conditions that produced the LYMErix failure have been addressed.

The hesitancy environment is materially worse than in 1998. Healthcare provider willingness to recommend Lyme borreliosis vaccination declined from 73% in 2018 to 68% in 2022, with safety concerns the dominant consideration [62]. The LYMErix narrative persists in clinical memory long after its evidential basis was dismantled, and anti-vaccine commentary in 2026 has already described VLA15 as using the same mechanism that caused the arthritis concerns with LYMErix, omitting the structural modification that defines its advance. The COVID-19 pandemic intensified VAERS-based reasoning in public discourse, and the social media infrastructure through which adverse event narratives propagate is substantially larger than in 1999 [63].

The Advisory Committee on Immunization Practices, whose routine recommendation will determine both the deployment of VLA15 at population level and its eligibility for Vaccine Injury Compensation Program coverage, underwent an unprecedented restructuring in June 2025 when all 17 members were replaced, with several appointees having publicly expressed skepticism toward established vaccine programmes. A federal court ruled in March 2026 that 13 of those appointees had been unlawfully installed [64]. Without a routine ACIP recommendation, VLA15 will face the same structural liability exposure that undermined LYMErix. The parallel with 1999 is direct, and the conditions are less favorable.

Implementation will require more than regulatory approval. It will require careful clinical communication, transparent discussion of the VALOR statistical limitations, and a targeted recommendation framework that limits vaccination to populations in whom individual benefit-risk clearly favours the intervention. Cost-effectiveness analyses have consistently found vaccination against Lyme borreliosis viable only at annual endemic infection probabilities above approximately 1% [65,66]. A recommendation broad enough to function as universal would enroll a substantial population in whom the individual case for vaccination is weak, making the benefit-risk asymmetry worse and the communication task harder. Compensation frameworks matter too: inclusion within national vaccine injury compensation schemes provides both legal structure and the symbolic reassurance that societies are prepared to support individuals in the rare event of serious adverse outcomes.

## 7. Conclusions

The lessons from LYMErix extend beyond Lyme borreliosis itself. This is not simply a story about *Borrelia*, ticks, or inflammatory arthritis. It is a case study on how scientific uncertainty, mechanistic plausibility, litigation, media narratives, and public trust interact within modern medicine. The medical community cannot assume that strong efficacy data alone will determine public acceptance. It cannot dismiss safety concerns simply because later evidence disproves them. Once a narrative becomes culturally embedded, it is extraordinarily difficult to reverse, as the persistence of the LYMErix story in clinical memory twenty-four years after the evidence base underlying it was refuted makes plain.

The re-emergence of a vaccine against Lyme borreliosis presents an opportunity not merely to prevent infection but to demonstrate that vaccinology has learned from its own history. Whether history repeats itself may depend less on the vaccine itself than on how the scientific and clinical community chooses to communicate its evidence.

## Data Availability

No new data were generated or analysed in support of this Perspective.
